# Interbasin trade worsens the state of freshwater fish biodiversity in China

**DOI:** 10.1016/j.isci.2024.111121

**Published:** 2024-10-09

**Authors:** Changbo Wang, E. Zhang, Yafei Wang, Yuan Chang, Pengpeng Zhang, Xiao Chen, Mingyue Pang, Han Yu, Qunwei Wang, Lixiao Zhang, Dequn Zhou, Manfred Lenzen, Arunima Malik, Donglan Zha, Xuejun Zhang, Meili Feng, Zhifu Mi

**Affiliations:** 1 College of Economics and Management & Research Center for Soft Energy Science, Nanjing University of Aeronautics and Astronautics, Nanjing 210016, China; 2 Laboratory of Digital Intelligence Management and Low-carbon Operations for Manufacturing System, Nanjing University of Aeronautics and Astronautics, Nanjing, China; 3 Institute of Hydrobiology, Chinese Academy of Sciences, Wuhan 430072, Hubei Province, P.R. China; 4 School of Statistics and Institute of National Accounts, Beijing Normal University, Beijing 100875, China; 5 School of Management Science and Engineering, Central University of Finance and Economics, Beijing 100081, China; 6 School of Geographical Sciences, Hebei Normal University, Shijiazhuang 050024, China; 7 College of Animal Science & Technology, Anhui Agricultural University, Hefei 230036, China; 8 Key Laboratory of Three Gorges Reservoir Region’s Eco-Environment, Ministry of Education, Chongqing University, Chongqing 400045, China; 9 Department of Natural Geography, Resources and Environment, Lanzhou University of Finance and Economic, Lanzhou 730101, China; 10 State Key Joint Laboratory of Environmental Simulation and Pollution Control, School of Environment, Beijing Normal University, Beijing 100875, China; 11 ISA, School of Physics A28, The University of Sydney, NSW 2006, Australia; 12 China Institute of Water Resources and Hydropower Research, Beijing 100038, China; 13 School of Geographical Sciences, Faculty of Science and Engineering, University of Nottingham Ningbo China, Ningbo 315100, China; 14 The Bartlett School of Sustainable Construction, University College London, London WC1E7HB, UK

**Keywords:** environmental science, environmental analysis, environmental resource, aquatic science, aquatic biology, economics

## Abstract

Human economic activities severely threaten freshwater fish biodiversity in different river basins. Trade makes the impact more mysterious and complex and confounds local efforts to protect freshwater biodiversity. To investigate the relationship between trade and freshwater fishes, we developed a river-basin economic transaction model that is applied to mainland China, home to 9% of the world’s freshwater fish species. Here, we show that interbasin trade induced by final demand contributes 74% of the threats to China’s freshwater fish biodiversity. Economically developed river basins (e.g., the Huaihe River) are the main beneficiaries of interbasin trade at the cost of biodiversity deterioration in economically underdeveloped river basins (e.g., the upper Pearl River), especially when trade occurs between distant basins. Our findings highlight the significance of the shift in governance from administrative divisions to river basins and control measures in different stages of economic supply chains to mitigate freshwater fish biodiversity threats.

## Highlights

• A multi-basin IO model is used for threats to fish biodiversity in China
• Interbasin trade induced by final demand contributes 74% of the threats
• Basins located in the upper reaches of rivers tended to be net exporters
• Economically developed basins benefit more from distant trade than adjacent trade

## Graphical abstract

**Figure:**
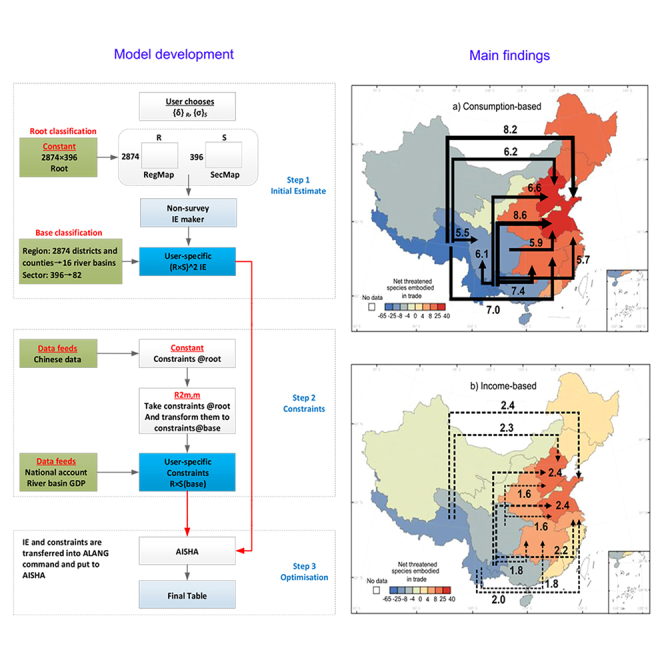

## Introduction

River basins are the spatial carriers of water resources and freshwater ecosystems. In addition, rivers support crucial economic and social activities of humans and serve as the habitats for diverse aquatic organisms, including 40% of global fish species.^1^ Freshwater fishes contribute to human welfare as key sources of food and income,^2^ and fish support the functioning and stability of ecosystems through their roles in the production of biomass and the regulation of trophic networks and nutrient cycles.^3^ However, human economic activities pose an increasingly intense threat to global freshwater fish biodiversity through direct resource exploitation, habitat encroachment, and environmental degradation.^4^^,^^5^^,^^6^^,^^7^^,^^8^^,^^9^ One of the main barriers to understanding the relationship between economic activities and threats to fish biodiversity lies in the spatial mismatch between the local generation of threats to biodiversity and the impacts due to interbasin trade.^10^^,^^11^^,^^12^^,^^13^ This means that the factors driving threats to fish biodiversity are partially attributable to both final consumption and primary inputs from outside the focal river basin.^14^ From a policy perspective, this spatial disconnect represents a substantial challenge to the assignment of responsibility for threats to fish biodiversity and the implementation of local protection policies.^15^

Previous studies have explored the environmental impacts (including threats to biodiversity) of spatial disconnection based on economic transactions between administrative units (e.g., countries or provinces).^16^^,^^17^^,^^18^^,^^19^^,^^20^^,^^21^^,^^22^ However, environmental impacts, especially those related to the hydrological and biogeochemical cycles (e.g., fish biodiversity and water resource management), are usually not restricted to administrative boundaries.^23^^,^^24^ Human activities rely on river basins to provide crucial natural resources, such as water and land, and hence alter basin-scale hydrological and biogeochemical cycles. This can threaten fish species distributed across surface water or groundwater networks of river basins.^25^^,^^26^ Regulating and addressing basin-scale environmental impacts by alleviating the adverse effects on freshwater fish biodiversity requires a shift in governance from administrative divisions to river basins. However, quantification of the environmental impacts caused by economic activities at the river basin scale is still lacking, as is disentangling the distribution and transfer of threats to freshwater fish biodiversity along the complex trade chains across river basins. Notably, such information is necessary to assign the responsibility for species conservation among economic participants.

This study addresses a critical research gap by examining the relationship between factors affecting freshwater fish biodiversity and economic activities at the river basin scale during different stages of the economic supply chain, including primary inputs, production, and final consumption. To determine effective control measures for biodiversity conservation, we developed a river-basin economic transaction model to investigate how threats to biodiversity shift along the interbasin supply chain. This approach will enable the government to identify whether threats to biodiversity within a river basin are derived from local or distant demand/investment. The analysis examines the impact of interbasin trade on biodiversity, highlighting the urgency of cross-river-basin collaboration for the protection of fish species and freshwater ecosystems.

We examined mainland China (hereafter referred to as China) as a case study. China has approximately 1,591 species of freshwater fish, representing about 9% of the global number of species; of these, 1,081 are endemic.^27^ The fishes are distributed among 16 river basins in China (Figure S1). Our focus on river basins is justified since the biodiversity of freshwater fishes in China is seriously threatened. The Chinese government has implemented a series of measures designed to protect freshwater ecosystems, including the river basin protection law and a 10-year fishing moratorium in the Yangtze River,^28^^,^^29^ and such efforts are expected to alleviate the deterioration of fish biodiversity. However, current policies overlook the impact of interbasin trade on aquatic ecosystems. Fish species in China are largely distributed in the central and western basins^30^ (Figure S2), regions that are also important agricultural and energy production centers and hence serve the entire country. Therefore, the impacts of interbasin trade on freshwater fish biodiversity in China must be considered when formulating protection measures.

In this study, we integrated information on threatened fish species with a river-basin economic transaction model. The implications of economic activities for fish biodiversity were based on 1,846 species/basin/threat records derived from China Red List of Freshwater Fishes.^27^ Based on a multi-basin input-output (IO) model, we distinguished geographical and sectoral factors (primary suppliers, direct threats, and final consumers) affecting threatened fish species in each river basin and evaluated the impacts of interbasin trade on freshwater fish biodiversity. We also examined the critical supply chain in terms of overall biodiversity impact and flagship species (e.g., *Acipenser sinensis* and *Psephurus gladius*). Our findings clarify the responsibility of different economic actors (producers, consumers, and investors) and support policy decisions in different stages of economic supply chains to protect the remaining endangered fish species and mitigate the degradation freshwater ecosystems.

## Results

### Contributions of geographical and sectoral sources

**Figure 1:**
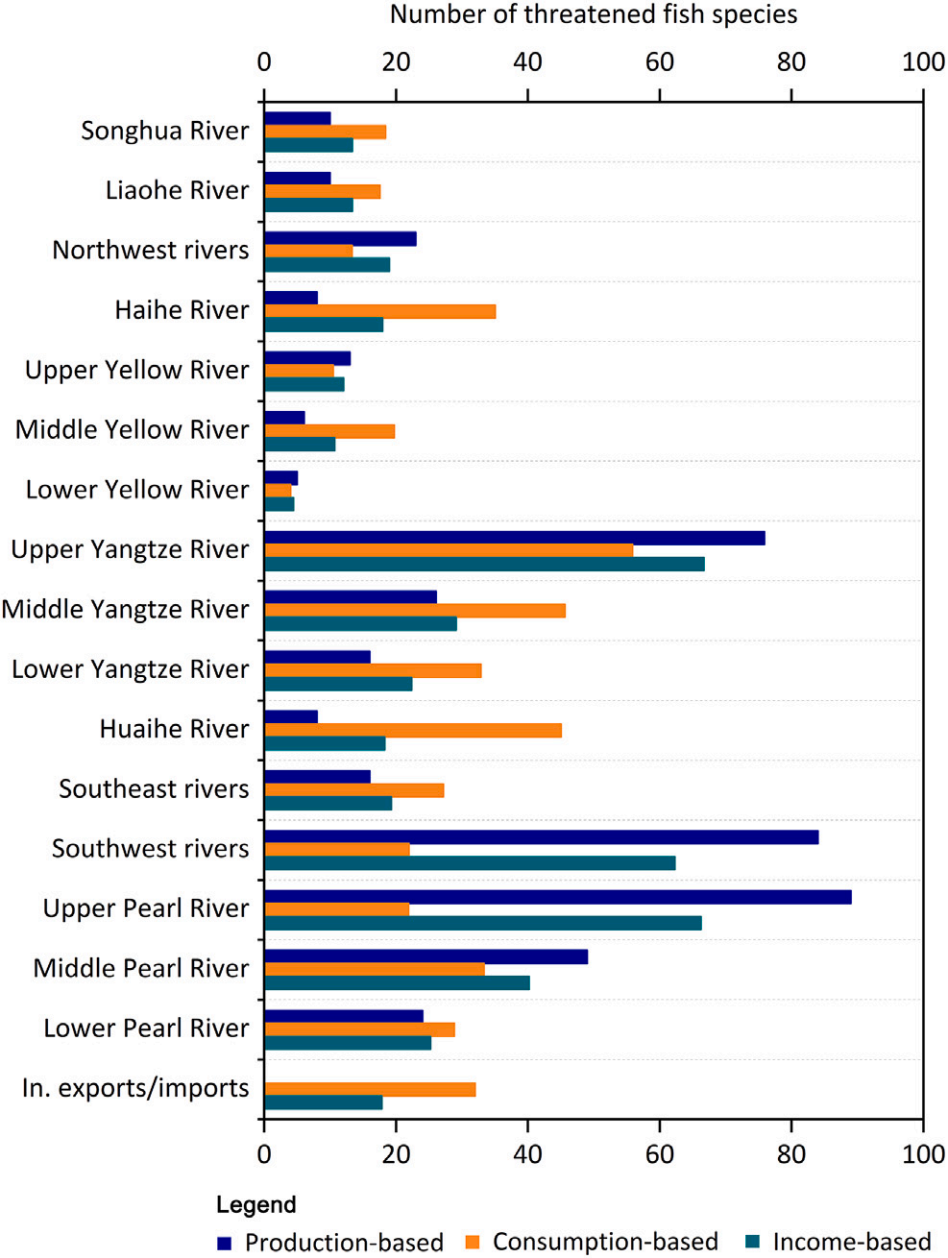
Number of threatened fish species in different river basins In. exports and In. imports represent international exports and international imports, respectively.

For geographical sources, the number of threatened fish species varied significantly among river basins (Figure 1). Economically less developed river basins were the major direct contributors to these nationally threatened species (e.g., the upper Pearl River, the Southwest Rivers, and the upper Yangtze River), while economically developed basins such as the Haihe River, the Huaihe River, and the mid-lower Yangtze River had higher consumption-based threats to biodiversity than those from the production- and income-based perspectives. No basins were dominated by income-based factors; the impacts from this perspective were always moderate, generally being more severe than consumption-based threats in economically underdeveloped basins and more severe than production-based threats in economically developed basins (Figure 1).

**Figure 2:**
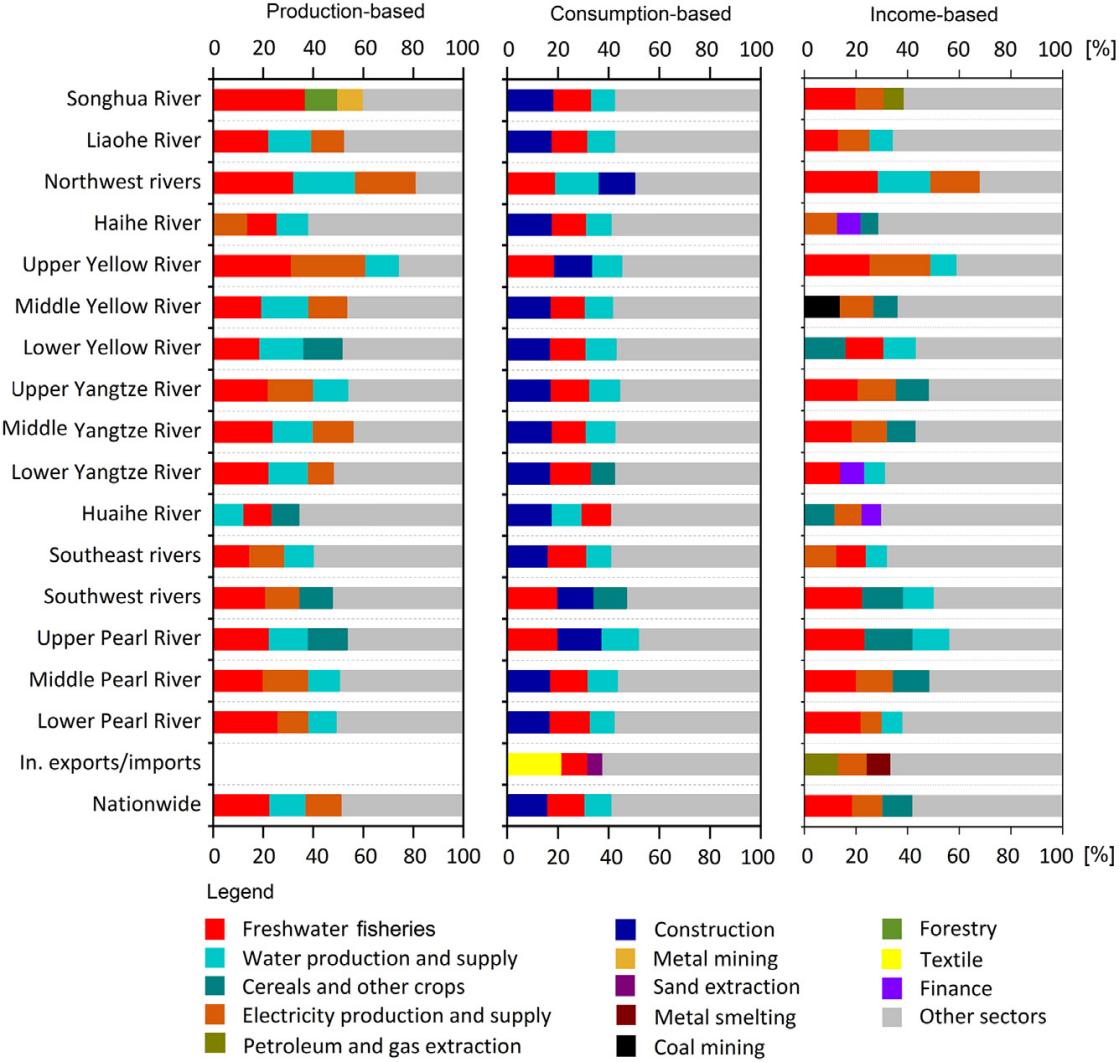
Sectoral contributions to fish biodiversity threats nationwide and in each river basin The sector names are shorthand versions of the standard names (see Table S1). Detailed results for the other sectors are shown in the supplemental data.

In terms of sectoral sources (Figure 2), freshwater fisheries, water production, and electricity generation comprised the primary direct threats to freshwater fish biodiversity nationwide, accounting for approximately 52% of the total. In particular, freshwater fisheries threatened the survival of fish through direct capture, bycatch, and the introduction of exotic species. Electricity generation, especially dam building for hydropower, blocked the migration routes of critical fish species (e.g., *Acipenser sinensis*) between river downstream floodplains and upstream tributaries. Water obtained through water intake engineering affected water quantity and quality and led to habitat degradation. From the consumption perspective, the final demand for products from the construction sector was the largest contributor, primarily due to rapid urbanization, followed by freshwater fisheries and water production. From the income perspective, primary inputs in freshwater fisheries, electricity generation, and grain planting were the main contributors.

For each river basin, we identified the top three sectoral sources from the production, consumption, and income perspectives and observed the respective distribution patterns (Figure 2). From the production perspective, freshwater fisheries were the most important threats to fish biodiversity for most basins, except the Haihe and Huaihe Rivers, where the largest contributors were electricity generation and water production, respectively. For the second largest factor, fish species from the upper reaches of the river basins were threatened by electricity generation (e.g., the upper Yangtze River, the upper Yellow River, and the Southwest Rivers), while those in the northern river basins were influenced by water production (e.g., the Liaohe River, the Northwest Rivers, and the middle and lower Yellow River). In addition, the contributions of cereal grains and other crops were significant in several river basins (e.g., the lower Yellow River, the Southwest Rivers, and the upper Pearl River). From the consumption perspective, construction was the most important factor for most river basins. However, freshwater fisheries were the largest contributors to the Northwest Rivers, the upper Yellow River, the Southwest Rivers, and the upper Pearl River. These findings indicated that economically developed basins comprised threats to biodiversity through a long causal chain, while economically less developed basins jeopardized fish biodiversity through direct effects (fishing). From the income perspective, the top three sources varied among river basins.

### Interbasin transfer of threats to freshwater fishes

**Figure 3:**
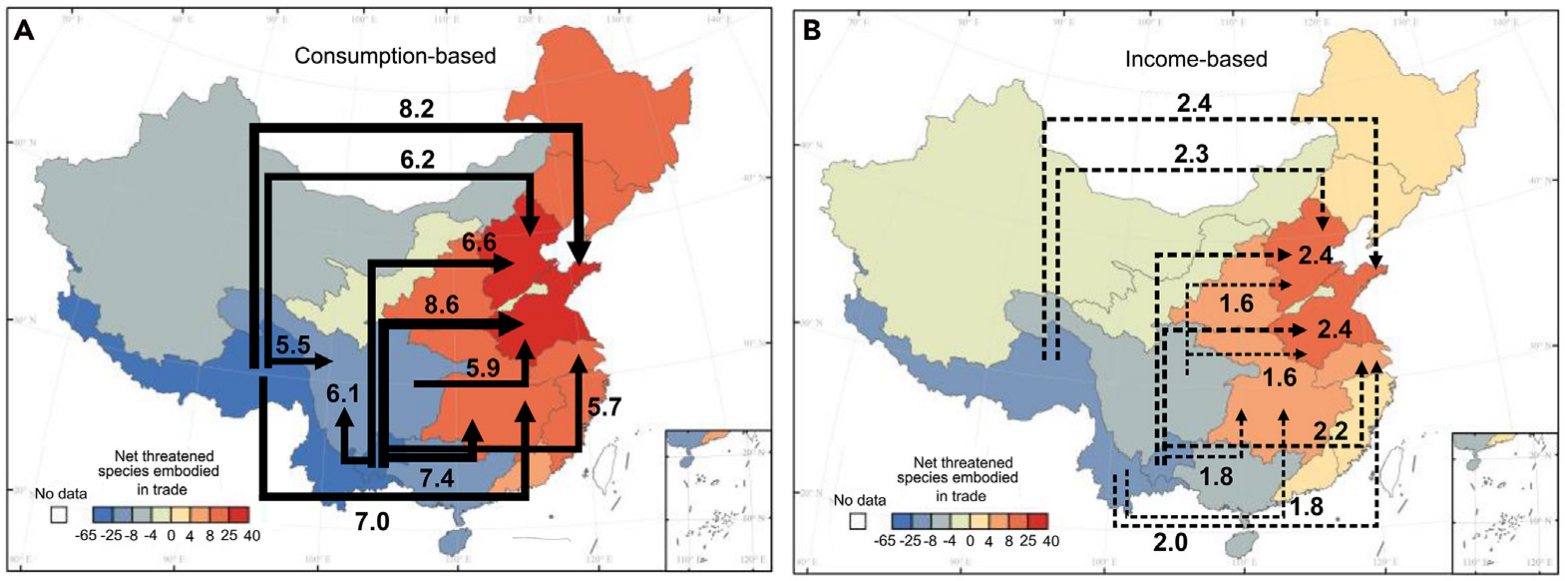
Ten largest fluxes of embodied freshwater fish biodiversity threats among the 16 river basins Ten largest fluxes of embodied freshwater fish biodiversity threats among the 16 river basins from the consumption (A) and income (B) perspectives. The shading of regions indicates the magnitude of net imports (reds) or net exports (blue) embodied in interbasin trade. Solid arrows represent fluxes from the consumption perspective, while dashed arrows represent fluxes from the income perspective.

Figure 3 shows the ten largest fluxes of threatened species induced by trade, i.e., the number of threatened species in a river basin that are embodied in goods and services exported to meet the final demand in other basins (consumption perspective) or those due to the primary inputs from other basins (income perspective). The shading of the basins in Figure 3 indicates the magnitude of net exports (blue) or net imports (red) embodied in interbasin trade.

From the consumption perspective, the threats to 74% of China’s freshwater fish species involved the production of goods and services that are ultimately consumed in different river basins in China or abroad. Basins located in the upper reaches of rivers and northwest China tended to be net exporters, while basins located in the mid-lower reaches of rivers and along the eastern coast (excluding the lower Yellow River) were almost all net importers of relevant commodities (Figure 3A and S8). This was probably due to the large number of threatened fish species distributed in upstream river basins and their associated stages in economic supply chains in China. For the nine net importers, 86% of their threatened species were linked to imports produced outside their boundaries. In contrast, the seven net exporters threatened fish biodiversity due to distant consumer demand. Among all net exporters, 76% of the threats to native fish species were linked to production for export. This proportion exceeded 80% in the upper Pearl River, the Southwest Rivers, and the upper and lower Yellow River (Figure S9). Further examination showed that the primary final destinations of biodiversity-implicated commodities were the Huaihe River, the Haihe River, the middle Yangtze River, and destinations abroad (Figure S10). The largest transfers of threats to biodiversity induced by final consumption were to the Huaihe River from the upper Pearl River (8.6 species), the Southwest Rivers (8.2 species), and the upper Yangtze River (5.9 species), and final demand in the middle Yangtze River was supported by substantial threats produced in the upper Pearl River (7.4 species) and the Southwest Rivers (7.0 species) (Figure 3A). Despite serving as net exporters, infrastructure construction required by the rapid urbanization of economically less developed river basins also had a significant impact on the fish species of the surrounding areas. For example, the final consumption in the upper Yangtze River was supported by factors threatening species in the upper Pearl River (6.1 species) and Southwest Rivers (5.5 species).

From the income perspective (Figure 3B), the cross-basin impact on freshwater fish was much weaker than the consumption aspect, with only 24% of the total threatened species affected by primary inputs from different river basins in China or abroad. The net importers and net exporters remained constant with the consumption-based results, although the ranking of several basins was slightly changed (Figure S10). Examining importers and exporters simultaneously showed that the general direction of the top ten fluxes of income-based biodiversity threats was similar to the consumption perspective but with a much narrower width (Figure 3B and S11).

Further examination of the sector content of these trade activities showed that supply chains threatening fish species primarily originated in economically underdeveloped river basins rich in fish biodiversity and with export-oriented energy and mineral industries (Tables S2 and S3). From the consumption perspective, the largest exports were destined for the construction sector in the Huaihe River. These were mostly electricity and building materials (soil, sand, and stone) from the Southwest Rivers and the upper Yangtze River. From the income perspective, primary inputs in the electricity generation sector in different basins stimulated the mutual growth of electricity products. The critical supply chains could also be traced for flagship species in each basin, such as *Acipenser sinensis* (Tables S4 and S5) and *Psephurus gladius* (Tables S6 and S7).

### Impacts of different trade scenarios

**Figure 4:**
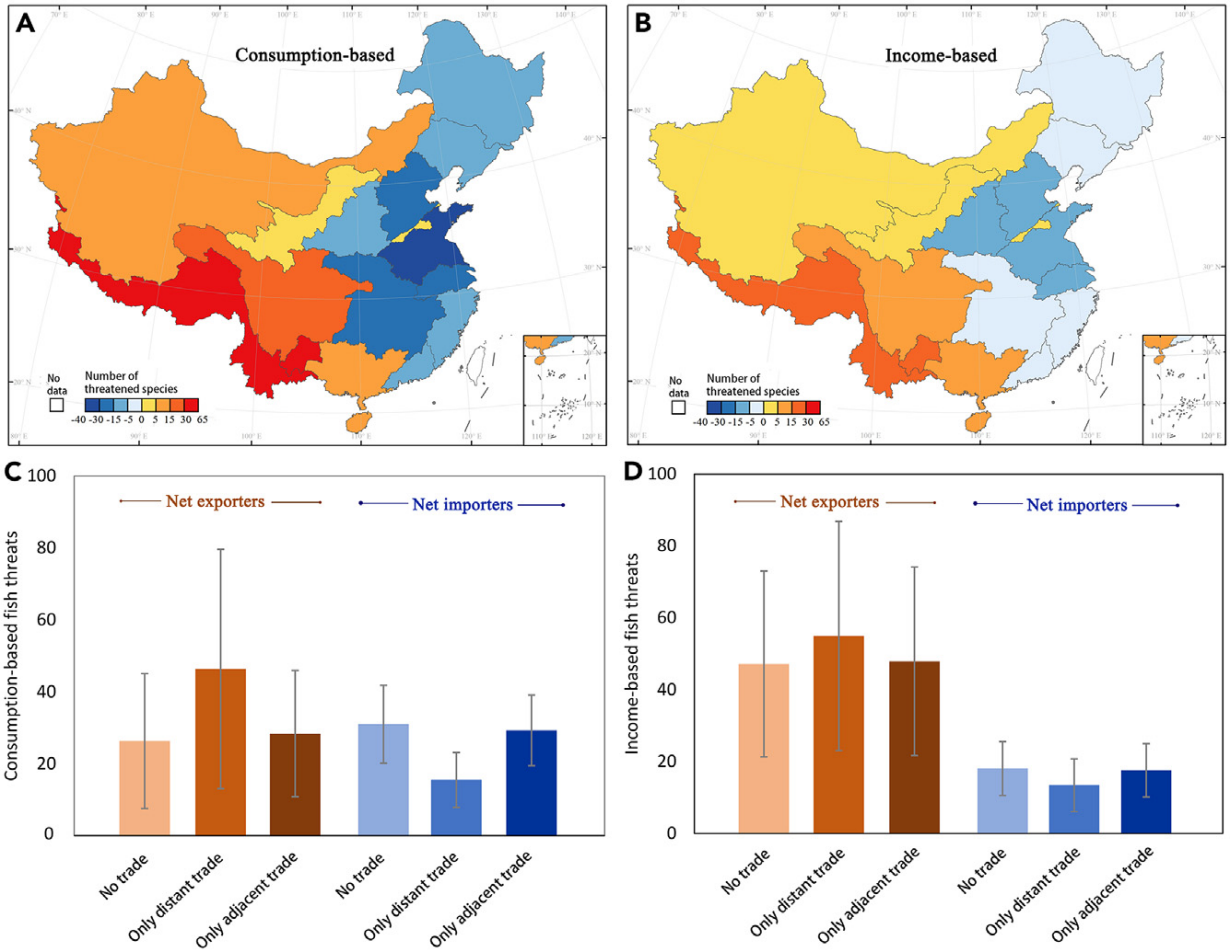
Impact of trade activities on freshwater fish biodiversity threats in different river basins (A and B) The impacts of trade on threatened species in different basins from consumption (A) and income (B) perspectives. The values were calculated from the difference in threatened species between the trade and no-trade scenarios at the basin level. (C and D) Threatened species for the original net importer and exporter basins under each trade scenario from the consumption (C) and income (D) perspectives. The error bars indicate the standard errors in the threatened species.

We considered a hypothetical scenario with the absence of interbasin trade for comparison with the existing interbasin trade to examine the impacts of trade on freshwater fish biodiversity in China. We assumed that trade partners produced the same goods and services that originally relied on interbasin trade.^31^ The impact of interbasin trade varied across river basins from both the consumption and income perspectives (Figure 4A and 4B). Generally, economically developed river basins located on the eastern coast (i.e., net importers under the existing trade scenario; see Figure S8) were the main beneficiaries of interbasin trade (Figure 4A), with consumption-based freshwater fish biodiversity threats being relieved for 6.35–37.60 species (17.19 species on average). Trade activities exacerbated freshwater fish biodiversity threats in economically less developed river basins (i.e., the net exporters under the existing trade scenario; see Figure S8) (Figure 4A), with the threatened species increasing by 0.65–61.35 (22.11 species on average). In particular, the Huaihe River and the upper Pearl River were the largest beneficiaries and victims, respectively. Interbasin trade from the income perspective had similar but much weaker impacts on freshwater fish biodiversity, with the variation in threatened species changing from −2.31 to −10.63 (net importers) and from 0.02 to 18.09 (net exporters) (Figure 4B).

Interestingly, the impacts of trade activities between adjacent basins (basins sharing land boundaries) and between distant basins (basins not sharing land boundaries) differed between net importer basins and net exporter basins (Figure 4C and 4D). On average, net importer basins experienced larger reductions from distant trade than from adjacent trade, while net exporter basins experienced greater increases in consumption/income-based threats from distant trade than from adjacent trade. Considering individual basins, several original net importer basins (e.g., the lower Yangtze River) would have slightly worse threats to biodiversity under the adjacent trade scenario, while the situation was the opposite for several original net exporter basins (e.g., the upper Yangtze River and the middle Pearl River) (Figures S12 and S13).

## Discussion

Our study has demonstrated that local threats to freshwater fish species are driven by consumer demand and primary inputs across different river basins. Therefore, policy decisions designed to support the remaining threatened fish species should consider the various stages of economic supply chains, i.e., the production side, demand side, and supply side.

From the production side, control measures such as preventing overfishing, developing eco-friendly water intake technology, and constructing fish passages in dammed rivers can be implemented to mitigate the direct damage to locally threatened freshwater fishes. Such measures may be applied to specific factors such as freshwater fisheries in the Yangtze River, electricity generation in the Haihe River, and water production in the Huaihe River. Increasing crop yields and minimizing farmland encroachment on riverbeds may be effective for the lower Yellow River, the Southwest Rivers, and the upper Pearl River because of the significant impact of crop cultivation.^32^^,^^33^ To reduce the damage caused by overfishing, a 10-year fishing moratorium was implemented in January 2021 with the goal of the recovery of fish stocks and aquatic biodiversity across the Yangtze River basin.^34^ However, final effects of this measure remain unclear, as overfishing is not the only important factor threatening aquatic ecosystems^35^ (Figure 2).

From the consumption side, control measures aim to guide green consumption behavior by imposing consumption taxes or promoting green labeling of implicated products. These demand-side measures can reduce the supply-chain implications of final consumption in critical geographical and sectoral sources such as the consumption of products from the construction sector in the mid-lower reaches and the eastern coast and products from freshwater fisheries in the upper reaches of river basins (Figure 2). From the consumption perspective, the fishing moratorium may have little effect on species protection, since the local demand for fishery products will shift to other river basins and thereby result in distant threats to biodiversity.

From the supply side, effective measures can control the downstream threats to biodiversity caused by primary inputs from critical geographical and sectoral sources. Decision makers must limit the investment in sectors with high levels of income-based threats to biodiversity such as agricultural production of cereals and other crops, coal mining, and freshwater fisheries. Different basins must adopt relevant effective measures according to the priority of the relevant stages of economic supply chains. For example, production-side control measures would be more effective than other measures in economically less developed basins such as the upper Yangtze River, the upper Pearl River, and the Southwest Rivers, while demand-side control measures would be more effective than other measures in economically developed basins such as the Haihe River, the Huaihe River, and the lower Yangtze River. No river basin is dominated by supply-side control measures (Figure 1).

The regional transfer of threats to freshwater fish biodiversity triggered by interbasin trade highlights the flow-based approach to mitigating the impacts of economic activities on fish species. Compared to traditional place-based governance, the flow-based approach considers an area in terms of its relationships with other areas by tracking and managing the origin, progress, and destination of key factors.^36^ Based on our results, control measures can be taken according to the relevant interbasin and intersectoral flows. Final demand basins, such as the Huaihe River, the Haihe River, and the middle Yangtze River can support their upstream basins (e.g., the upper Pearl River and the Southwest Rivers) to reduce direct threats through technical and financial transfer. Furthermore, final demand sectors such as construction in the Huaihe River should endeavor to reduce the threats to biodiversity from upstream sectors (e.g., electricity generation in the Southwest Rivers and sand extraction in the upper Yangtze River) by providing financial support or by supervising the production process.

Our results indicated that distant trade has a stronger effect on freshwater fish biodiversity in river basins than adjacent trade.^31^ One contributing factor is that basins usually have more distant trade partners than adjacent basins, resulting in a higher frequency of trade between distant basins than between adjacent basins. Furthermore, the similar socioeconomic and environmental conditions of adjacent basins limit the trade of products and services and hence the concomitant impacts. In contrast, distant basins usually have a greater disparity in natural resources and economic structure and thus allow basins to diversify imported goods and services to make full use of comparative advantages. Future research on the environmental impacts of interbasin trade should differentiate the effects of trade with distant basins from those with nearby basins to identify the most important socioeconomic and environmental factors. Our results also suggest that future exploration of river basin protection measures must consider environmental spillover effects such as the threats to freshwater fish biodiversity caused by interbasin trade. Because economically developed basins (net importers) tend to displace biodiversity threats to economically less developed basins (net exporters), policies can set consumption (income)-based targets that assign the responsibility for these threats to consumers (suppliers) instead of only to producers.

There is an international context to our findings. Several authors have noted that there is the potential for synergies between freshwater fish conservation and UN Sustainable Development Goals (SDGs)^1^^,^^37^; these include the targets for No Poverty (SDG 1), Zero Hunger (SDG 2), Clean Water and Sanitation (SDG 6), Responsible Consumption and Production (SDG 12), and Life on Land (SDG 15). Freshwater fisheries provide critical sources of food and shelter to low-income populations in economically less developed basins (SDG 1 and 2). Meanwhile, a bidirectional relationship exists between freshwater fish biodiversity and No Poverty (SDG 1). Notably, sustainable freshwater fisheries can promote fishing activity and thus contribute to poverty alleviation and income growth.^38^ However, agriculture and small hydropower generation are the economic pillars in the basins located in the mid-upper reaches of rivers,^39^ providing evitable food and affordable electricity but destroying fish habitats and altering aquatic ecosystem functions.^40^^,^^41^ In addition, upstream river basins are important reservoirs of fish biodiversity and drinking water sources in China. Although this has not been adequately addressed, protecting aquatic ecosystem functions for freshwater fish can improve water quality for human use (SDG 6).^42^^,^^43^ As an important component of terrestrial ecosystems, fish species play key roles in food webs and ecosystem functioning (SDG 15).^3^ Recognizing these synergistic or bidirectional relationships is critical for policy decisions designed for the coordinated development of freshwater fish biodiversity conservation and the associated SDGs.

This study represents an attempt to build an interbasin economic transaction model that facilitates the analysis of environmental impacts at the basin scale. We suggest the transition of governance from administrative divisions to river basins to avoid the “silo effects” due to inter-jurisdictional fragmentation.^23^ The river chief system in China can be considered an improvement compared to the traditional administration, but it only focuses on single rivers, not larger basins.^44^ Appropriate organizations must be established to address important basin-scale issues such as freshwater fish biodiversity conservation. With the development of an environmental database including more detailed threat factors and the IO model with higher sectoral resolution, the impacts of trade on freshwater ecosystems can be further explored, along with more practicable and effective control measures. The framework presented in this study could also be applied to the driving factors of other river-basin-scale environmental issues (e.g., water resource pressure, water pollution, and flood/drought disasters) in different stages of economic supply chains.

### Limitations of the study

Sources of uncertainty within this study included modeling interbasin trade and linking the causes of biodiversity threats to various economic activities. The multi-basin IO table employed in this study was compiled in the Chinese Industrial Ecology Virtual Laboratory (Chinese IELab), in which multiregional input-output (MRIO) standard deviations were calculated by fitting an error propagation formula to the standard deviations of the raw data points.^16^^,^^45^ The standard deviations of MRIO data are visualized in Figure S7, and we found that large table elements (increasing along the logarithmic horizontal axis) had better data reliability than small elements. We linked freshwater fish biodiversity threats to economic activities using the approach proposed by Lenzen et al.,^46^ which was also adopted in subsequent studies.^14^^,^^47^ We acknowledged that the relationships between threats to freshwater fish biodiversity and human activities are not binary variables, but rather continuous measures. To improve the accuracy of the satellite account, we considered the distribution proportion and the intensity of human impacts for individual fish species instead of using equal weighting.

## Resource availability

### Lead contact

Further information and requests for resources and reagents should be directed to and will be fulfilled by the lead contact, Qunwei Wang (wqw0305@126.com).

### Materials availability

This study did not generate any new physical materials.

### Data and code availability

• This paper analyzes existing, publicly available data that are listed in the key resources table. The results of the data generated during the analysis can be found in Data S1 and S2.
• Code for the analysis is written in MATLAB and is available from the lead contact upon reasonable request.
• Any additional information required to reanalyze the data reported in this paper is available from the lead contact upon reasonable request.

## Acknowledgments

We are grateful for financial support from the 10.13039/501100001809National Natural Science Foundation of China (grant no. 52225902, 72373064, 72161147003, and 31572234), the Humanities and Social Sciences Foundation of the Ministry of Education (grant no. 22YJCZH184), and the Major Project of Philosophy and Social Science Research in Colleges and Universities of Jiangsu Province (grant no. 2022SJZD050).

## Author contributions

C.W., L.Z., Q.W., and M.L. designed the research; E.Z., X.Z., Y.C., X.C., and M.F. contributed the data; C.W. developed the models and conducted the simulations with support from Y.W., Y.C., P.Z., M.P., and Z.M.; and C.W., Y.C., and H.Y. wrote the manuscript with contributions from D. Zhou, A.M., D. Zha, and Z.M. All authors reviewed and commented on the manuscript.

## Declaration of interests

The authors declare no competing interests.

## STAR★Methods

### Key resources table

**Table:** 

REAGENT or RESOURCE	SOURCE	IDENTIFIER
**Deposited data**		

The Chinese multibasin input‒output table for 2017 of 16 basins and 82 sectors	This paper	https://data.mendeley.com/datasets/jj8r76yt3h/1

**Software and algorithms**		

MATLAB	MathWorks	https://www.mathworks.com

### Method details

#### Process of compiling the multi-basin input-output table

We constructed a multi-basin input-output (IO) table based on the Chinese Industrial Ecology Virtual Laboratory (Chinese IELab) following the method outlined in ref. 16 and 45. The model included domestic monetary transactions between 82 economic sectors and final consumers (households and government) across 10 river basins in China. We further divided each of the Yangtze River, the Yellow River, and the Pearl River basins into three subbasins (upper, middle, and lower reaches) and generated an IO table containing 16 river basins. The base year was 2017.

Multi-regional input-output (MRIO) tables built in Chinese IELab are based on the ‘root-base’ construction principle. The “root” here means the most detailed region and sector classification in Chinese IELab, while “base” indicates the user-specific region and sector classification that required by specific research target. The root classification in Chinese IELab includes 396 commodity groups and 2874 districts/counties without Taiwan, Hong Kong, and Macau.

As shown in Figure S3, there are three steps to compile the multi-basin input-output table. At the initial estimate step (step 1), the base classification is defined as 16 river basins and 82 sectors. This study focuses on the relationship between economic activities in different river basins and fish biodiversity, thus river basin is selected as regional unit. The 82 sectors are selected according to the threat records in China’s Red List of Freshwater Fishes^27^ and we retain the detailed sectors that directly threaten freshwater fishes, such as cultivation of cereals and other crops, freshwater fishery, electricity production and supply, water production and supply, and construction (see Table S1). We map all districts/counties (root) to river basins (base) by using a root-to-base concordance matrix (2874 × 16). We assign a value of 1 to those districts/counties that corresponded directly to each river basin, and 0 otherwise. Although most districts/counties will belong to a certain river basin, the boundaries of river basins do not completely coincide with the districts/counties (see Figure S4). Therefore, we correspond them to different river basins according to the location of the district/county governments. Similarly, we aggregate the 396 sectors into 82 sectors with a sectoral root-to-base concordance matrix (396 × 82).

To make the multi-basin IO table more reliable and accurate, we must impose some constraints on the compiling process (step 2). Constraints are external data that are added to the optimization operation in order to refine the initial estimate. Here we use the national gross domestic product (GDP) (national account) and GDP in each river basins (river basin GDP) as constraints to make the data value in the input-output table close to the real economic data.

Finally, all data files are transferred to an Automated Integration System for Harmonised Accounts (AISHA) to reconcile the base input-output table using advanced construction workflows (step 3). Figure S5 shows the final multi-basin input-output table in basic prices generated from the Chinese IELab. To verify the accuracy of the table, we compare the final demand value of each river basin (equivalent to GDP calculated by expenditure method) and real GDP of each river basin, and find these two figures were close, with an average difference of 15.6% (see Figure S6).

#### Creating a satellite account of fish biodiversity impacts

There are 351 threatened freshwater fish species in China Red List of Freshwater Fishes,^27^ classified into five levels of threat (critical risk, vulnerability, endangered, extinction, and regional extinction). We excluded the extinction and regional extinction categories, and the remaining dataset available for our analysis included 348 species. We followed the approach explained by Lenzen et al.^46^ to construct our freshwater fish biodiversity indicator, a method that was also adopted in two recent studies.^14^^,^^48^ The dataset included the geographical distributions of 348 freshwater fishes and the major threat factors affecting each species.^27^ The latter corresponded to the 166 standard factors included in the IUCN Red List.^49^

A schematic representation of the process used to construct the biodiversity indicators is shown in Figure S14. The first step was to establish the relationship between each of the IUCN threat factors and the 82 economic sectors within the multi-basin IO table. We considered 166 human factors from the 197 in the IUCN list, with the remaining natural factors omitted. The links between 166 human factors and 82 economic sectors were established based on the IUCN documentation.^49^ Following the method and nomenclature of Lenzen et al.,^46^ we constructed a binary K × C concordance matrix **B**_**1**_. We assigned a value of 1 to industrial sectors that directly corresponded to each threat and 0 otherwise. Here, K = 166 IUCN threats, and C = 82 economic sectors. Matrix **B**_**1**_ was post-multiplied by C × T concordance matrices **B**_**2**_^**(c)**^ that related sectors to river basins. Here, T = 1312 economic sectors (16 basins and 82 sectors in each basin). This yielded 16 K × T binary concordance matrices **B**^**(c)**^ linking IUCN threats to sectors in the 16 river basins. An example for three K × T binary concordance matrices **B**^**(c)**^ is given in Table S8 (assuming K = 4 threat causes and T = 15 economic sectors).

The second step was to normalize **B**^**(c)**^ to **N**^**(c)**^ using two different weighting schemes. The CO_2_ emissions for each sector were adopted for the threat due to climate change, while the remaining sectors were normalized based on their gross industrial output. The normalization resulted in the row sum of the concordance matrix **N**^**(c)**^ equaling 1; this prevented multiple counting of IUCN threats. Table S9 provides an example of this normalization step.

The third step was to establish a link between basin/species/cause records and economic sectors. We constructed a binary S × K concordance matrix **S** linking the basin/species/cause records and 166 IUCN threats. Here, S = 1846 basin/species/cause records, derived from China Red List of Freshwater Fishes.^27^ Then, we obtained the concordance matrix **C = SN**^**(c)**^, that describes the relationship between each entry of species/basin/cause within the China Red List of Freshwater Fishes database and the corresponding basin/sector represented in **N**^**(c)**^. An example of a **C** matrix is shown in Table S10. Matrix **C** was then aggregated by referring to the same basin/species record to create an R × T matrix **C**_**ag**_ (see the example in Table S11). Here, R = 455 basin/species. The row sums of **C**_**ag**_ equal the number of causes listed for each record. The matrix **R** was then obtained by normalizing **C**_**ag**_. Following the method described in Lenzen et al.,^46^ all threat causes were weighted equally to obtain **R**. We applied this method by considering the distribution area of individual species in different river basins and the intensity of interference from human economic activities when weighting **C**_**ag**_.^14^^,^^48^ The Global Human Influence Index calculated based on human population pressure (population density), human land use, and infrastructure (e.g., built-up areas and night-time lights), and human access (e.g., roads and navigable rivers) is widely used to reflect the intensity of human influence.^14^^,^^50^ For this study, we assumed that an individual threat to a fish species was a function of the distribution area of the species in each river basin and the intensity of human influence within the basin (i.e., the Global Human Influence Index). Finally, all threats to individual species were normalized to 1 (matrix **R**). An example of an **R** matrix is provided in Table S12.

The final step was to use the aggregated matrix **R** based on species identity to create a new matrix **R**_**ag**_ that described species threats against exerting sectors and therefore conformed to the standard format required for an EE-MRIO (environmental extended multiregional input-output) analysis. An example of an **R**_**ag**_ matrix is given in Table S12.

#### EE-MRIO analysis

The extended multiregional input-output model was developed by integrating the sectoral direct biodiversity threats and the multi-basin IO table describing the exchange of product within and among river basins (Table S1). The EE-MRIO model tracks fish biodiversity threats from the basin of final consumption (the final consumers) to the basin of production (the direct threats) through product supply chains, and it also tracks fish biodiversity threats from the basin of primary inputs (the primary suppliers) to the basin of production (the direct threats) through product sale chains.

The production-based, consumption-based, and income-based fish biodiversity threats can be measured using Equations 1, 2, and 3, respectively:

Equation (1) $$P_{r}={\phi^{\prime }X}_{r}$$

Equation (2) $$C_{r}=\phi^{\prime }LY_{r}$$

Equation (3) $$S_{r}=V_{r}G\phi$$

where $P_{r}$, $C_{r}$, and $S_{r}$ indicate basin- and sector-specific production-based, consumption-based, and income-based fish biodiversity threats, respectively. The column vector ${\phi =R}_{ag}{\hat{X}}^{-1}$ refers to the direct biodiversity threats for a unitary output of sectors. $X_{r}$ indicates the total output of sector *r* or each sector in region *r*. The notation ‘'’ indicates the transposition of the vector ϕ. The column vector $Y_{r}$ represents the final demand of sector *r* or region *r*. The row vector $V_{r}$ represents the primary input in sector *r* or region *r*. ***L*** is the Leontief inverse ${\left(I-T\hat{X}\right)}^{-1}$ of the multi-basin IO table ***T*** that captures both direct and indirect inputs from various sectors to produce the unitary final demand of specific sectors. ***G*** is the Ghosh inverse ${\left(I-{\hat{X}}^{-1}T\right)}^{-1}$ that captures both direct and indirect outputs from various sectors enabled by the unitary primary input of specific sectors.

The basic input‒output model can be employed to quantify the biodiversity threats embodied in trade. For example, the biodiversity threats in basin *i* caused by the final demand of sector or basin *j* can be calculated as follows:

Equation (4) $$C_{ij}=\phi_{i}^{\prime }LY_{j}$$

The biodiversity threats in basin *i* enabled by primary inputs from sector *j* or basin *j* can be calculated as follows:

Equation (5) $$S_{ij}=V_{j}G\phi_{i}$$

where $\phi_{i}$ denotes a vector with the direct biodiversity threat intensity for basin *i* but zero for other basins; $Y_{j}$ represents the final demand of sector *j* or basin *j*, and $V_{j}$ represents the primary input in sector *j* or basin *j*.

### Quantification and statistical analysis

There are no quantification or statistical analyses to include in this study.

## Supplemental information

Document S1. Figures S1–S14 and Tables S1–S12

Data S1 (separate file). The number of threatened freshwater fishes contributed by each sector of different river basins from diverse accounting perspectives (production-, consumption-, and income based), related to Figure 2

Data S2 (separate file). Data used to create the figures in the manuscript, related to Figure 1, 2, 3, and 4
