# Supplementary material for: Interbasin trade worsens the state of freshwater fish biodiversity in China
Source: iScience. 2024 Oct 9;27(11):111121. doi: 10.1016/j.isci.2024.111121 (PMC11539590; doi:10.1016/j.isci.2024.111121)
Supplement: Document S1. Figures S1–S14 and Tables S1–S12 [file mmc1.pdf]

iScience, Volume *27*

Supplemental information

Interbasin trade worsens the state

# of freshwater fish biodiversity in China

Changbo Wang, E. Zhang, Yafei Wang, Yuan Chang, Pengpeng Zhang, Xiao Chen, Mingyue Pang, Han Yu, Qunwei Wang, Lixiao Zhang, Dequn Zhou, Manfred Lenzen, Arunima Malik, Donglan Zha, Xuejun Zhang, Meili Feng, and Zhifu Mi

Supplemental Figures:

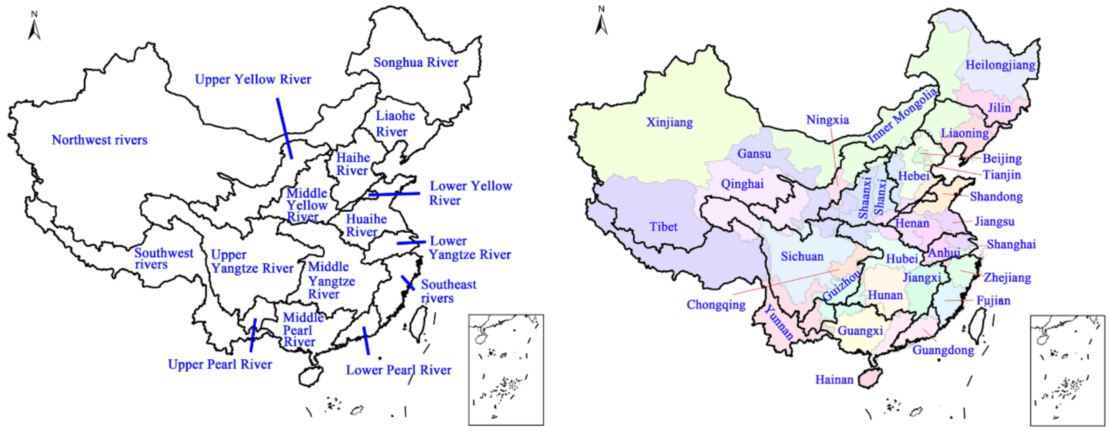

Figure S1. Geographical boundaries for sixteen river basins. The Yangtze River, Yellow River and Pearl River are divided into the upper, middle and lower reaches.

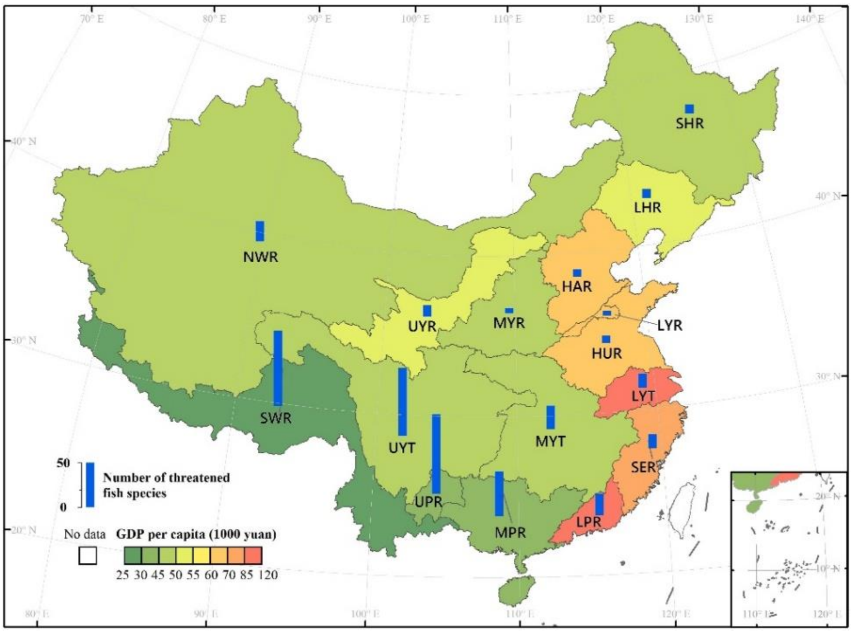

Figure S2. Spatial distributions of threatened freshwater fish species in China. SHR, Songhua River; LHR, Liaohe River; NWR, Northwest Rivers; HAR, Haihe River; HUR, Huaihe River; UYR, Upper Yellow River; MYR, Middle Yellow River; LYR, Lower Yellow River; UYT, Upper Yangtze River; MYT, Middle Yangtze River; LYT, Lower Yangtze River; SER, Southeast Rivers; SWR, Southwest Rivers; UPR, Upper Pearl River; MPR, Middle Pearl River; LPR, Lower Pearl River.

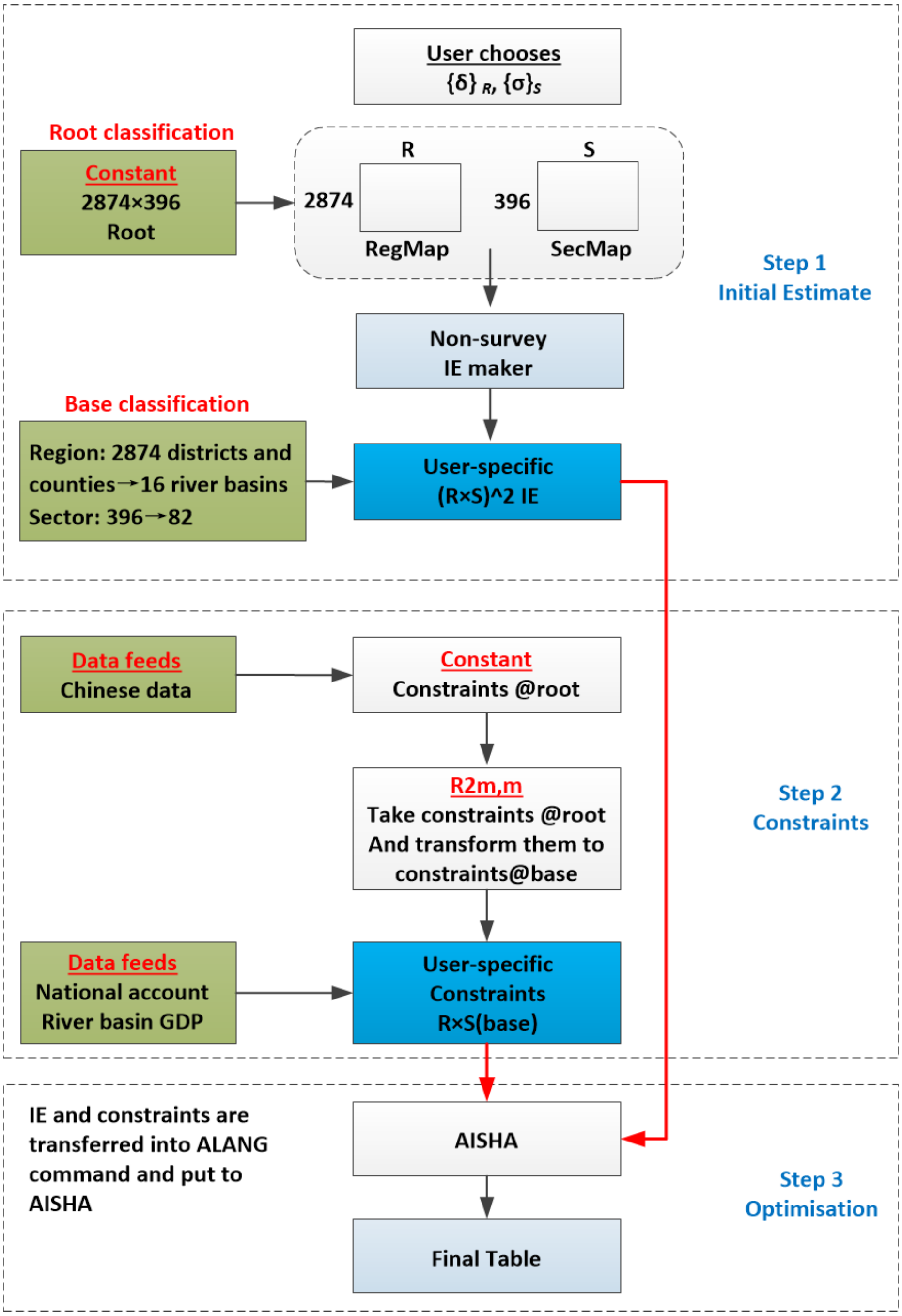

Figure S3. Schematic of the construction process of the Chinese multi-basin input-output model.

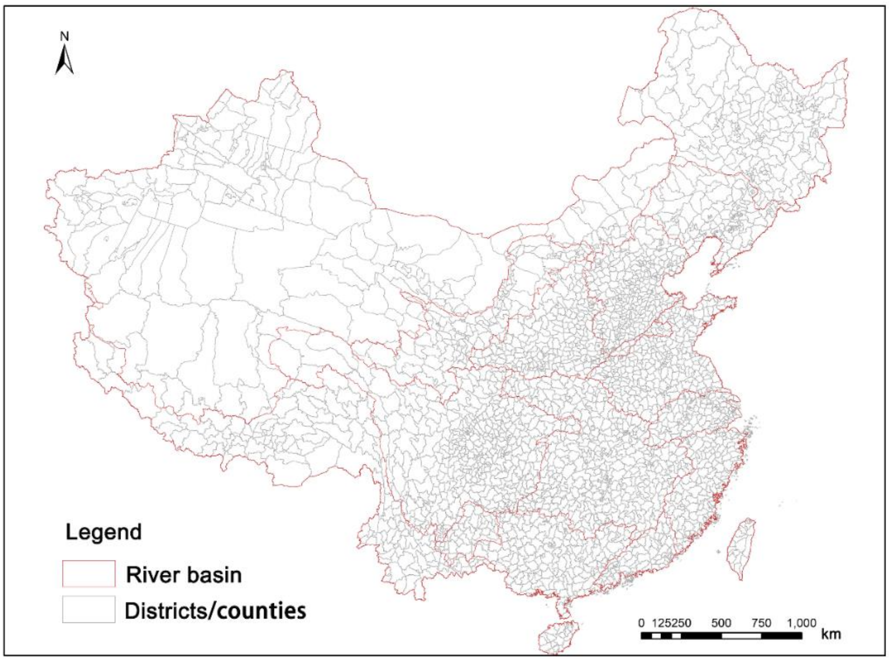

Figure S4. Comparison of geographical boundary between river basins and districts/counties.

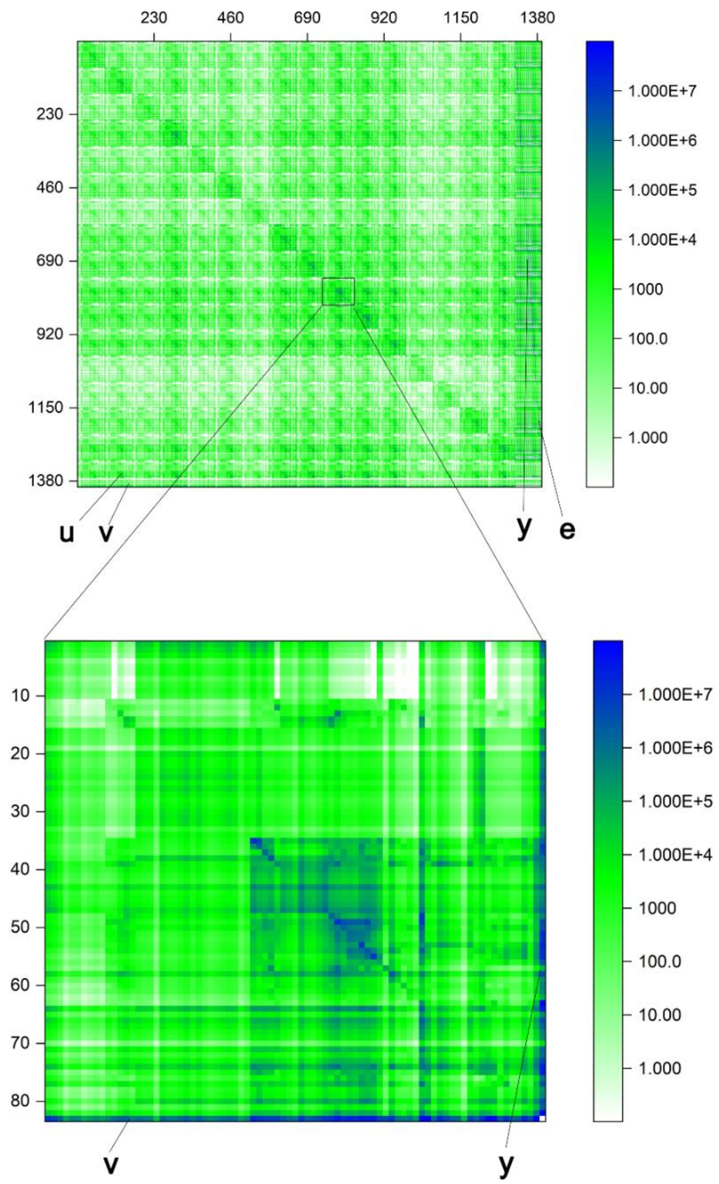

Figure S5. Heat map of the Chinese multi-basin IO table (top-view) and the lower Yangtze River section of the IO table (zoomed up bottom-view). x- and y-axes show sector numbers. The complete multi-basin IO table measures 1398×1393, whereas the lower Yangtze River section of the table has (82+4)×(82+5) = 86×87 sectors. Grey shades represent the transaction values expressed in 10^4 Chinese yuan. **v**, value added; **y**, domestic final demand; **u**, imports matrix; **e**, exports vector.

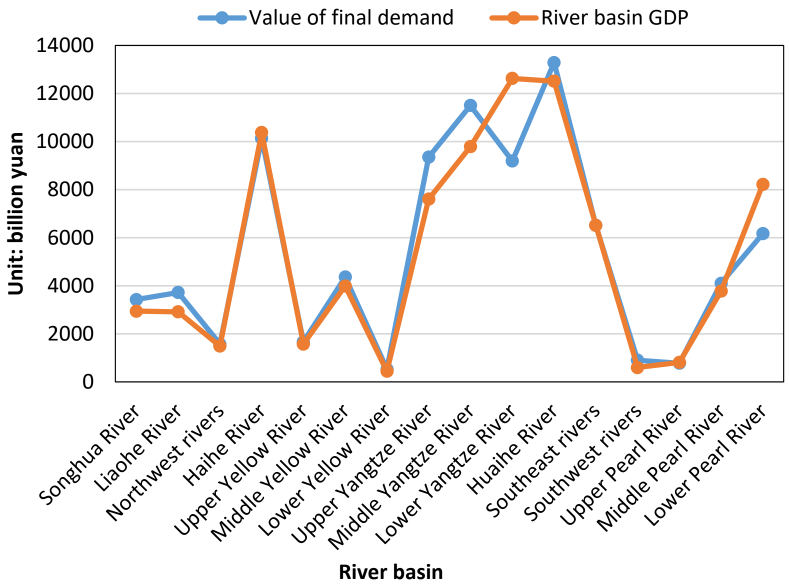

Figure S6. Comparison between the value of final demand and real GDP in each river basin.

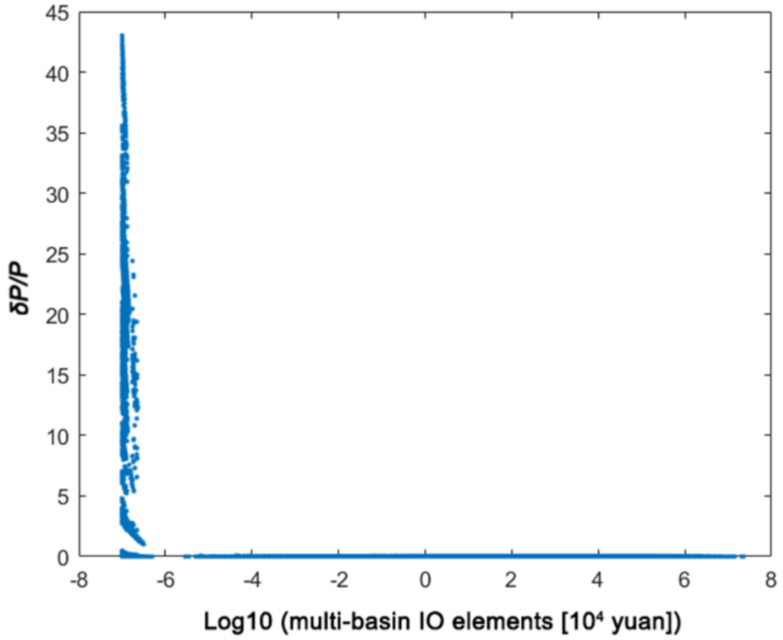

Figure S7. Relative standard deviations $\sigma P/P$ of Chinese multi-basin input-output (IO) data P. The base year was selected as 2017. This table has a good data reliability because large table elements are adequately supported by raw data points, and these elements are relatively stable under KRAS balancing and thus have low uncertainty.

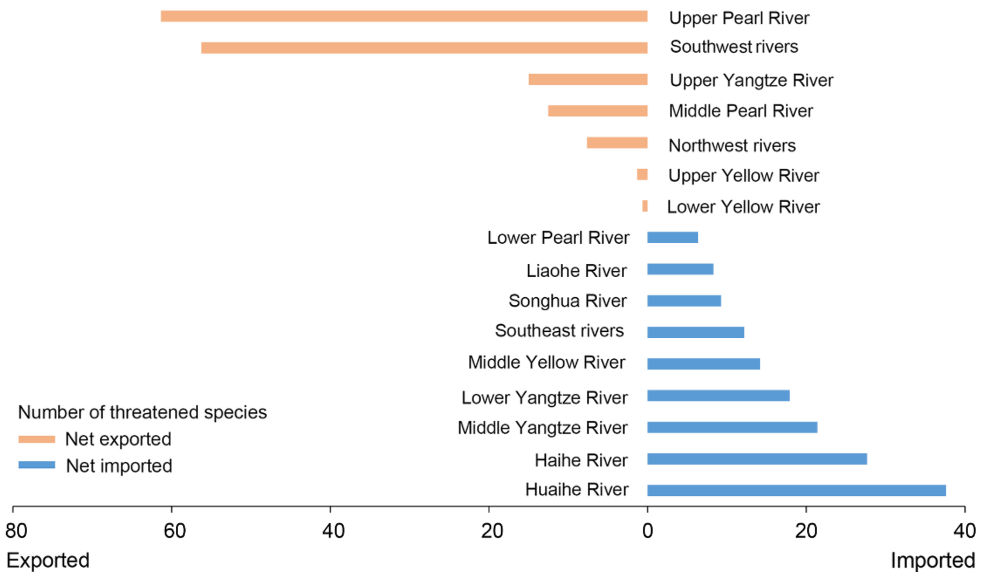

Figure S8. Net importers and exporters of inland fish biodiversity threats from the consumption-based perspective.

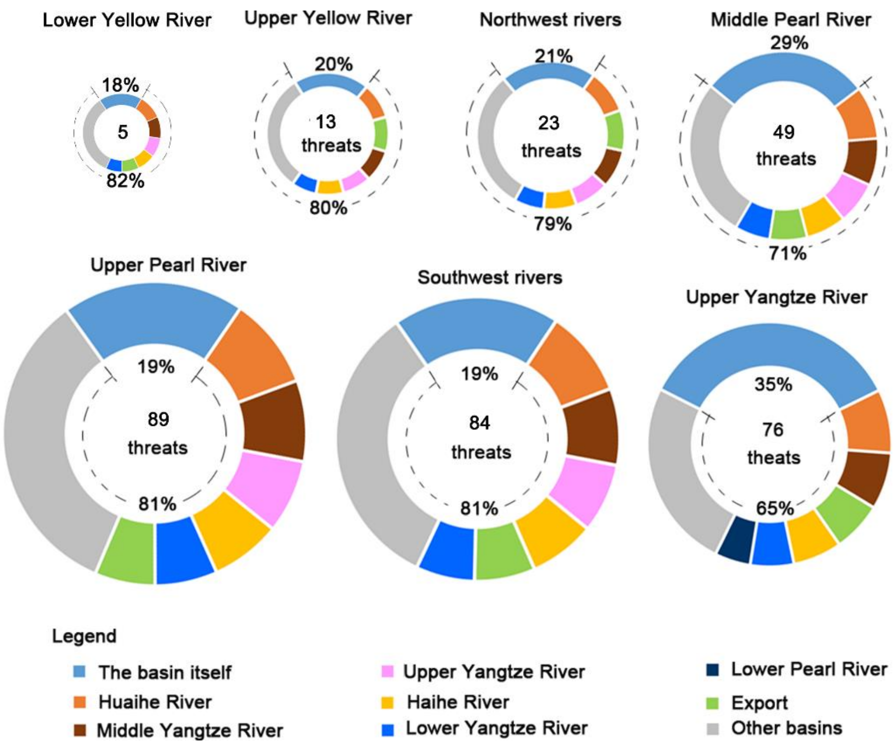

Figure S9. Seven net exporters and final destinations of biodiversity-implicated commodities from the consumption-based perspective.

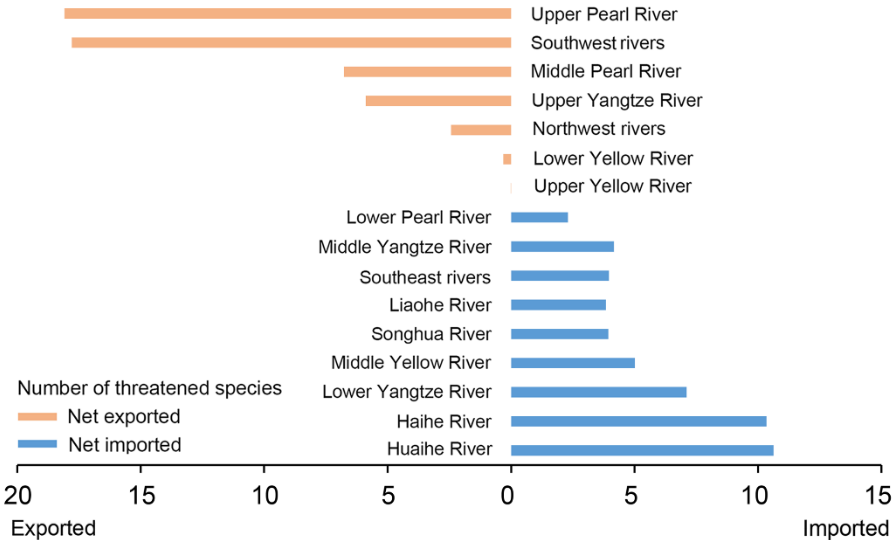

Figure S10. Net importers and exporters of inland fish biodiversity threats from the income-based perspective.

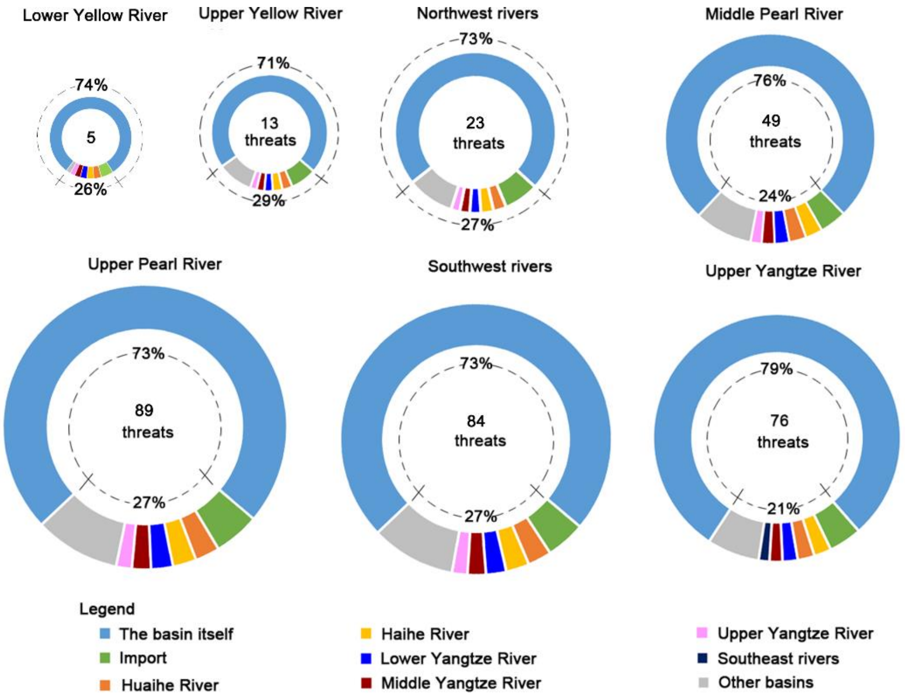

Figure S11. Seven net exporters and primary suppliers of biodiversity-implicated commodities from the income-based perspective.

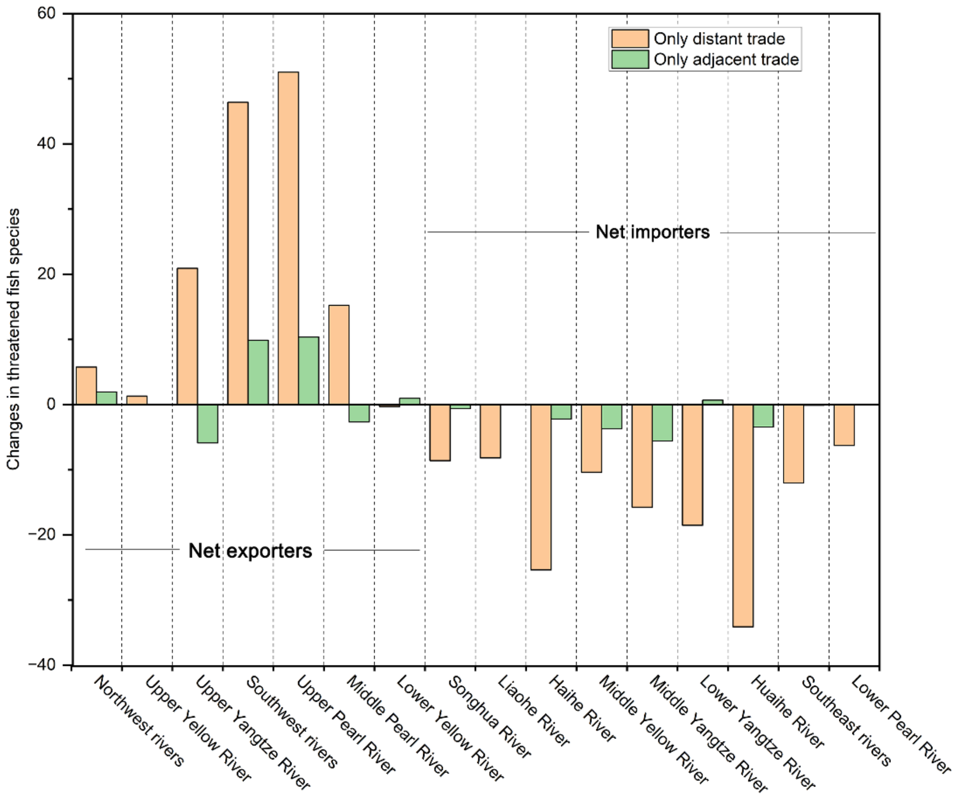

Figure S12. Threatened species for the original net importer and exporter basins under the only distant and adjacent trade scenario from consumption perspective.

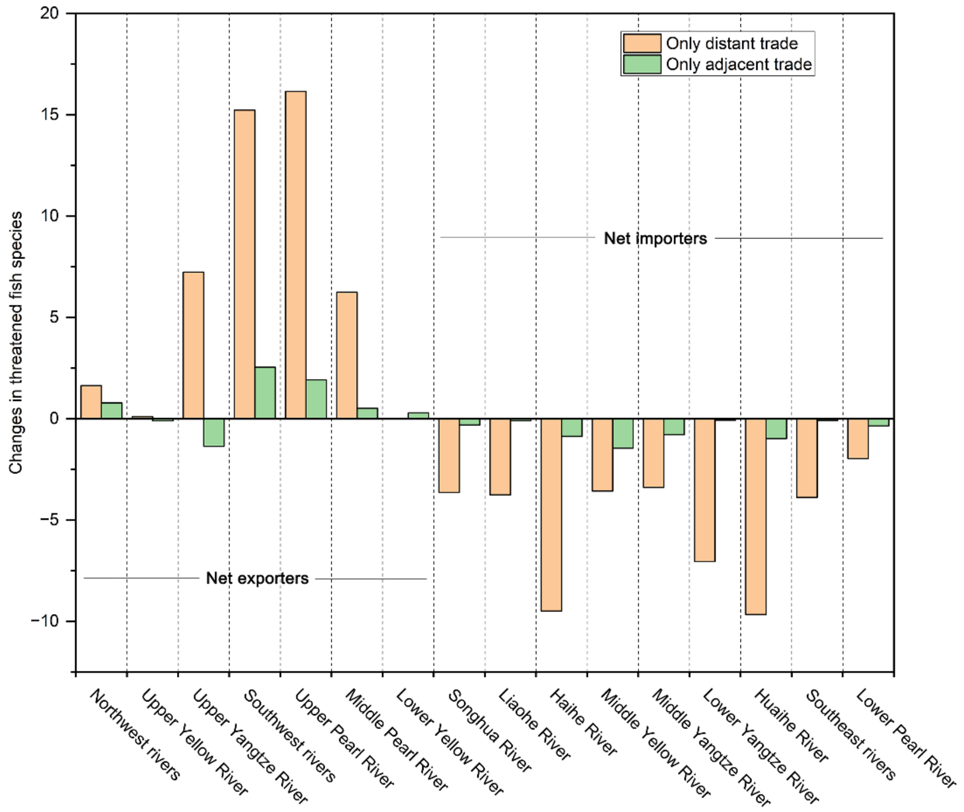

Figure S13. Threatened species for the original net importer and exporter basins under the only distant and adjacent trade scenario from income perspective.

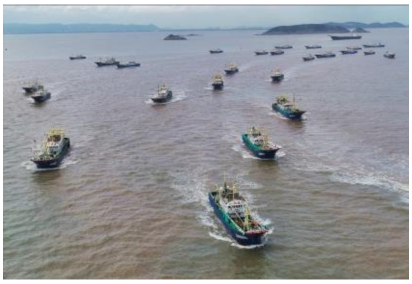

STEP 1: Threats to the species are extracted from the standard IUCN Threat Classification. Concordance matrix $\mathbf{B_1}$ is constructed linking IUCN threats to economic sectors within the multi-basin IO table. For example IUCN Threat "Fishing & harvesting aquatic resources" is linked to sector "Freshwater fishing". Concordance matrix B1 is multiplied to produce matrix $\mathbf{B}^{(c)}$ describing the link between IUCN threats and corresponding sectors for each of the 16 basins. Matrix $\mathbf{B}^{(c)}$ is weighted by economic output (except for climate change where CO_2 emission are used) and normalised to produce matrix $\mathbf{N}^{(c)}$ where row total equal 1 to avoid double counting threats . As IUCN threat categories correspond to multiple sectors in the multi-basin IO, economic weighting adjust the contribution of the sectors assuming that larger sectors exert more pressure on Inland fish species.

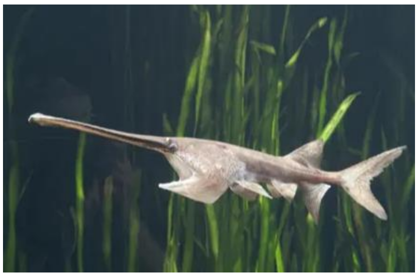

STEP 2: IUCN individual species records are linked to the corresponding entries in $\mathbf{N}^{(c)}$. The resultant matrix **C** describes the relationship between each individual entry of species/basin/threat within the IUCN database and the corresponding basin/ sector represented in $\mathbf{N}^{(c)}$. Our example is based on the *Psephurus gladius* listed as Critically Endangered in the IUCN Red List. The IUCN threat "Fishing & harvesting aquatic resources" links to the corresponding entry in $\mathbf{N}^{(c)}$ representing the "Freshwater fishing" Sector in The multi-basin IO table.

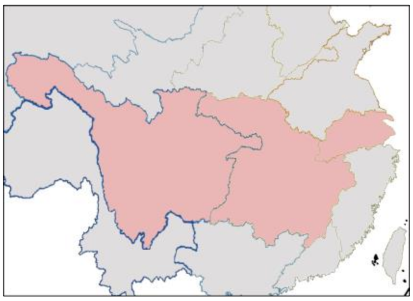

STEP 3: The species is distributed across three river basins, i.e., the upper, middle and lower Yangtze River as shown. Matrix **C** is therefore constructed based on the corresponding entries in $\mathbf{N}^{(c)}$ for these three basins and the one "Freshwater fishing" sector. Matrix **C** is then aggregated by entries referring to the same basin/species record to create matrix $\mathbf{C}_{ag}$. Rows within this matrix refer to basin/species and columns the corresponding basin/sectors to which threats are attributed.

STEP 4: Data is processed to consider the species range and human influence. In the example of the *Psephurus gladius*, 50% of the range is within the middle Yangtze River, 29% within the upper Yangtze River, and 21% the lower Yangtze River.

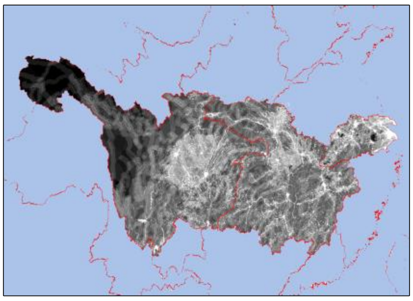

STEP 5: We calculate a mean value for the level of human influence index within each river basin where the species is distributed. In our example for the middle Yangtze River this is 19.7, the upper Yangtze River 15.1, and for the lower Yangtze River 23.6. The matrix $\mathbf{C}_{ag}$ is then weighted as a function of the range of species (step 4) by human influence, standardised to 1 to produce matrix **R**. In our example for the *Psephurus gladius* at the end of this process, the 1 IUCN Threat "Fishing & harvesting aquatic resources" is attributed to the "Freshwater fishing" sector with 0.52 of the threat associated with the middle Yangtze River, 0.22 with the upper Yangtze River, and 0.26 with the lower Yangtze River. Finally, matrix **R** is aggregated on species identity to produce matrix $\mathbf{R}_{ag}$ which describes species threats against exerting sectors.

Figure S14. Schematic representation of steps taken in construction of the biodiversity indicator within the extended multi-basin input-output model.

Supplemental Tables:

Table S1. Sector classification of the Chinese multi-basin input-output table

Sector index	Sector names	Shorthand versions
1	Cultivation of cereals and other crops	Cereals and other crops
2	Cultivation of vegetables and horticultural crops	Vegetables
3	Cultivation of fruits, nuts, beverages and spice crops	Fruits
4	Cultivation of traditional Chinese medicine	Traditional Chinese medicine
5	Forestry	Forestry
6	Animal feeding	Animal feeding
7	Other animal husbandry	Other animal husbandry
8	Marine fishing	Marine fishing
9	Freshwater fishery	Freshwater fishery
10	Agriculture, forestry, animal husbandry and Fishery Services	Agriculture Services
11	Coal mining and beneficiation products	Coal mining
12	Oil and gas production products	Oil and gas
13	Metal ore mining and dressing products	Metal ore
14	Soil, sand and stone extraction	Sand extraction
15	Non metallic ore and other ore mining and dressing products	Non metallic ore
16	Grain milling	Grain milling
17	Feed processing	Feed processing
18	Vegetable oil processing	Vegetable oil
19	Sugar refining	Sugar
20	Slaughtering and meat processing	Slaughtering
21	Aquatic products processing	Aquatic products
22	Vegetable, fruit and nut processing	Vegetable processing
23	Other agricultural and sideline food processing	Other agricultural food
24	Baked goods manufacturing	Baked goods
25	Candy, chocolate and preserves manufacturing	Candy
26	Instant food manufacturing	Instant food
27	Manufacturing of liquid milk and dairy products	Dairy products
28	Canning	Canning
29	Manufacturing of condiments and fermented products	Condiments
30	Other food manufacturing	Other food
31	Alcohol	Alcohol
32	Soft drink manufacturing	Soft drink
33	Refined tea processing	Refined tea
34	Tobacco	Tobacco
35	Textile	Textile
36	Textile clothing, shoes, hats, leather, down and their products	Textile clothing
37	Wood processing products and furniture	Wood products
38	Paper making, printing, cultural, educational and sporting goods	Paper making

Table S1 (continue). Sector classification of the Chinese multi-basin input-output table

Sector index	Sector names	Shorthand versions
39	Petroleum, coking products and nuclear fuel processing products	Petroleum, coking and nuclear
40	Basic chemical raw material manufacturing	Basic chemicals
41	Fertilizer manufacturing	Fertilizer
42	Pesticide manufacturing	Pesticide
43	Other chemical products	Other chemicals
44	Pharmaceutical products	Pharmaceutical products
45	Chemical fiber products	Chemical fiber products
46	Rubber products	Rubber products
47	Plastic products	Plastic products
48	Non metallic mineral products	Non metallic mineral products
49	Metal smelting and calendering products	Metal smelting
50	Metal products	Metal products
51	General equipment	General equipment
52	Special equipment	Special equipment
53	Transportation equipment	Transportation equipment
54	Electrical machinery and equipment	Electrical equipment
55	Communication equipment, computers and other electronic equipment	Communication equipment
56	Other manufactured products	Other manufactured products
57	Scrap	Scrap
58	Electricity production and supply	Electricity production
59	Heat production and supply	Heat production
60	Gas production and supply	Gas production
61	Water production and supply	Water production
62	Sewage disposal	Sewage disposal
63	Construction	Construction
64	Wholesale and retail	Wholesale and retail
65	Railway transportation	Railway transportation
66	Road transportation	Road transportation
67	Urban public transportion	Urban public transportion
68	Waterway transportation	Waterway transportation
69	Air transportation	Air transportation
70	Pipeline transportation	Pipeline transportation
71	Handling and storage	Handling and storage
72	Accommodation	Accommodation
73	Catering	Catering
74	Finance	Finance
75	Real estate	Real estate

Table S1 (continue). Sector classification of the Chinese multi-basin input-output table

Sector index	Sector names	Shorthand versions
76	Scientific research	Scientific research
77	Technical service	Technical service
78	Leasing	Leasing
79	Travel agency	Travel agency
80	Business services	Business services
81	Recreation	Recreation
82	Other services	Other services

Table S2. Top-ranking inter-basin industrial path causing threats to species from consumption perspective

No.	# of threats	Path
1	0.04	Southwest rivers electricity production industry → Final demand in Huaihe River in construction sector
2	0.04	Upper Yangtze River electricity production industry → Final demand in Huaihe River in construction sector
3	0.04	Upper Yangtze River sand extraction industry → Final demand in Huaihe River in construction sector
4	0.03	Southwest rivers sand extraction industry → Final demand in Huaihe River in construction sector
5	0.03	Southwest rivers electricity production industry → Final demand in upper Yangtze River in construction sector
6	0.03	Middle Pearl River electricity production industry → Final demand in Huaihe River in construction sector
7	0.03	Upper Pearl River sand extraction industry → Final demand in Huaihe River in construction sector
8	0.03	Upper Yangtze River electricity production industry → Final demand in lower Yangtze River in construction sector
9	0.03	Southwest rivers electricity production industry → Final demand in lower Yangtze River in construction sector
10	0.03	Southwest rivers electricity production industry → Final demand in upper Yangtze River in electricity production sector

Table S3. Top-ranking inter-basin industrial path causing threats to species from income perspective

No.	# of threats	Path
1	0.08	Primary inputs in middle Yangtze River in electricity production sector → Upper Yangtze River electricity production industry
2	0.07	Primary inputs in middle Yangtze River in electricity production sector → Middle Pearl River electricity production industry
3	0.07	Primary inputs in upper Yangtze River in electricity production sector → Middle Pearl River electricity production industry
4	0.04	Primary inputs in middle Pearl River in electricity production sector → Upper Yangtze River electricity production industry
5	0.03	Primary inputs in middle Yangtze River in electricity production sector → Upper Yellow River electricity production industry
6	0.03	Primary inputs in upper Yangtze River in electricity production sector → Upper Yellow River electricity production industry
7	0.03	Primary inputs in upper Yangtze River in electricity production sector → Middle Yangtze River electricity production industry
8	0.03	Primary inputs in Northwest rivers in electricity production sector → Upper Yangtze River electricity production industry
9	0.03	Primary inputs in middle Yangtze River in electricity production sector → Lower Pearl River electricity production industry
10	0.03	Primary inputs in upper Yangtze River in electricity production sector → Lower Pearl River electricity production industry

Table S4. Top-ranking industrial path causing threats to flagship species (*Acipenser sinensis*) from consumption perspective

No.	# of threats	Path
1	0.0061	Middle Yangtze River waterway transportation industry → Final demand in middle Yangtze River in construction sector
2	0.0030	Middle Yangtze River water production industry → Final demand in middle Yangtze River in other service sector
3	0.0028	Middle Yangtze River air transportation industry → Final demand in middle Yangtze River in construction sector
4	0.0026	Middle Yangtze River water production industry → Final demand in middle Yangtze River in construction sector
5	0.0017	Middle Yangtze River non metallic mineral product industry → Final demand in middle Yangtze River in construction sector
6	0.0012	Middle Yangtze River inland fishing industry → Final demand in middle Yangtze River in cereals and other crops sector
7	0.0009	Middle Yangtze River waterway transportation industry → Final demand in Huaihe River in construction sector
8	0.0009	Middle Yangtze River water production industry → Final demand in middle Yangtze River in transportation equipment sector
9	0.0009	Middle Yangtze River cereals and other crops industry → Final demand in middle Yangtze River in cereals and other crops sector
10	0.0008	Middle Yangtze River inland fishing industry → Export of product from middle Yangtze River textile sector

Table S5. Top-ranking industrial path causing threats to flagship species (*Acipenser sinensis*) from income perspective

No.	# of threats	Path
1	0.0022	Primary inputs in middle Yangtze River in cereals and other crops sector → Middle Yangtze River cereals and other crops industry
2	0.0017	Primary inputs in middle Yangtze River in non metallic mineral product sector → Middle Yangtze River construction industry
3	0.0015	Primary inputs in middle Yangtze River in water production sector → Middle Yangtze River water production industry
4	0.0006	Primary inputs in middle Yangtze River in metal smelting sector → Middle Yangtze River construction industry
5	0.0006	Primary inputs in middle Yangtze River in road transportation sector → Middle Yangtze River construction industry
6	0.0005	Primary inputs in middle Yangtze River in wholesale and retail sector → Middle Yangtze River construction industry
7	0.0004	Primary inputs in middle Yangtze River in metal smelting sector → Middle Yangtze River metal smelting industry
8	0.0004	Primary inputs in middle Yangtze River in electricity production sector → Middle Yangtze River electricity production industry
9	0.0004	Primary inputs in middle Yangtze River in wholesale and retail sector → Middle Yangtze River cereals and other crops industry
10	0.0003	Primary inputs in middle Yangtze River in communication equipment sector → Middle Yangtze River communication equipment industry

Table S6. Top-ranking industrial path causing threats to flagship species (*Psephurus gladius*) from consumption perspective

No.	# of threats	Path
1	0.0030	Middle Yangtze River water production industry → Final demand in middle Yangtze River in other service sector
2	0.0027	Upper Yangtze River water production industry → Final demand in upper Yangtze River in other service sector
3	0.0026	Lower Yangtze River water production industry → Final demand in lower Yangtze River in other service sector
4	0.0026	Middle Yangtze River water production industry → Final demand in middle Yangtze River in construction sector
5	0.0025	Lower Yangtze River water production industry → Final demand in lower Yangtze River in construction sector
6	0.0023	Upper Yangtze River water production industry → Final demand in upper Yangtze River in construction sector
7	0.0020	Middle Yellow River water production industry → Final demand in middle Yellow River in other service sector
8	0.0019	Middle Yellow River water production industry → Final demand in middle Yellow River in construction sector
9	0.0017	Middle Yangtze River non metallic mineral product industry → Final demand in middle Yangtze River in construction sector
10	0.0016	Upper Yellow River water production industry → Final demand in upper Yellow River in other service sector

Table S7. Top-ranking industrial path causing threats to flagship species (*Psephurus gladius*) from income perspective

No.	# of threats	Path
1	0.0023	Primary inputs in upper Yangtze River in cereals and other crops sector → Upper Yangtze River cereals and other crops industry
2	0.0022	Primary inputs in middle Yangtze River in cereals and other crops sector → Middle Yangtze River cereals and other crops industry
3	0.0021	Primary inputs in middle Yellow River in cereals and other crops sector → Middle Yellow River cereals and other crops industry
4	0.0017	Primary inputs in lower Yangtze River in water production sector → Lower Yangtze River water production industry
5	0.0016	Primary inputs in upper Yellow River in cereals and other crops sector → Upper Yellow River cereals and other crops industry
6	0.0015	Primary inputs in middle Yangtze River in water production sector → Middle Yangtze River water production industry
7	0.0014	Primary inputs in lower Yellow River in cereals and other crops sector → Lower Yellow River cereals and other crops industry
8	0.0013	Primary inputs in middle Yellow River in water production sector → Middle Yellow River water production industry
9	0.0013	Primary inputs in upper Yellow River in water production sector → Upper Yellow River water production industry
10	0.0012	Primary inputs in upper Yangtze River in water production sector → Upper Yangtze River water production industry

Table S8. Numerical example for three river basins' $K \times T$ binary concordance Matrices $\mathbf{B}^{(c)}$, each listing K=4 threat causes, and T=15 industry sectors.

$\mathbf{B}^{(c)}$ matrix, binary concordance	Basin 1					Basin 2					Basin 3				
	Grain	Fishing	Electricity	Industry	Service	Grain	Fishing	Electricity	Industry	Service	Grain	Fishing	Electricity	Industry	Service
Basin 1 Overfishing	0	1	0	0	0	0	0	0	0	0	0	0	0	0	0
Basin 1 Dam	0	0	1	0	0	0	0	0	0	0	0	0	0	0	0
Basin 1 Water pollution	1	1	1	1	1	0	0	0	0	0	0	0	0	0	0
Basin 1 Climate change	1	1	1	1	1	1	1	1	1	1	1	1	1	1	1
Basin 2 Overfishing	0	0	0	0	0	0	1	0	0	0	0	0	0	0	0
Basin 2 Dam	0	0	0	0	0	0	0	1	0	0	0	0	0	0	0
Basin 2 Water pollution	0	0	0	0	0	1	1	1	1	1	0	0	0	0	0
Basin 2 Climate change	1	1	1	1	1	1	1	1	1	1	1	1	1	1	1
Basin 3 Overfishing	0	0	0	0	0	0	0	0	0	0	0	1	0	0	0
Basin 3 Dam	0	0	0	0	0	0	0	0	0	0	0	0	1	0	0
Basin 3 Water pollution	0	0	0	0	0	0	0	0	0	0	1	1	1	1	1
Basin 3 Climate change	1	1	1	1	1	1	1	1	1	1	1	1	1	1	1

Table S9. Numerical example for three river basins' $K \times T$ normalised concordance Matrices $\mathbf{N}^{(c)}$, each listing K=4 threat causes, and T=15 industry sectors..

$\mathbf{N}^{(c)}$ matrix, binary concordance	Basin 1					Basin 2					Basin 3				
	Grain	Fishing	Electricity	Industry	Service	Grain	Fishing	Electricity	Industry	Service	Grain	Fishing	Electricity	Industry	Service
Basin 1 Overfishing	0	1	0	0	0	0	0	0	0	0	0	0	0	0	0
Basin 1 Dam	0	0	1	0	0	0	0	0	0	0	0	0	0	0	0
Basin 1 Water pollution	0	0	0.56	0.42	0.03	0	0	0	0	0	0	0	0	0	0
Basin 1 Climate change	0.02	0.01	0.17	0.07	0.03	0.01	0.01	0.10	0.05	0.03	0.03	0.01	0.07	0.34	0.05
Basin 2 Overfishing	0	0	0	0	0	0	1	0	0	0	0	0	0	0	0
Basin 2 Dam		0	0	0	0	0	0	1	0	0	0	0	0	0	0
Basin 2 Water pollution	0	0	0	0	0	0	0	0.59	0.18	0.24	0	0	0	0	0
Basin 2 Climate change	0.02	0.01	0.17	0.07	0.03	0.01	0.01	0.10	0.05	0.03	0.03	0.01	0.07	0.34	0.05
Basin 3 Overfishing	0	0	0	0	0	0	0	0	0	0	0	1	0	0	0
Basin 3 Dam	0	0	0	0	0	0	0	0	0	0	0	0	1	0	0
Basin 3 Water pollution	0	0	0	0	0	0	0	0	0	0	0	0	0.29	0.57	0.14
Basin 3 Climate change	0.02	0.01	0.17	0.07	0.03	0.01	0.01	0.10	0.05	0.03	0.03	0.01	0.07	0.34	0.05

**Weighting variables**															
Sectoral CO_2 emissions (Mt)	50	15	500	200	100	40	15	300	150	80	100	30	200	1000	150
Sectoral gross output (Mt)	10	5	20	15	1	50	25	100	30	40	20	10	50	100	25

Table S10. Numerical example for matrix **C**, following the example given in **Tabs S8.1** and **S8.2**. The elements of matrices **C** sum up to *S* basin/species/cause sub-records.

**C** matrix, weighting of each cause	Basin 1					Basin 2					Basin 3				
Total: 9 species-basin-cause sub-records	Grain	Fishing	Electricity	Industry	Service	Grain	Fishing	Electricity	Industry	Service	Grain	Fishing	Electricity	Industry	Service
Species A, Basin 1, overfishing	0	1	0	0	0	0	0	0	0	0	0	0	0	0	0
Species A, Basin 2, climate change	0.02	0.01	0.17	0.07	0.03	0.01	0.01	0.10	0.05	0.03	0.03	0.01	0.07	0.34	0.05
Species A, Basin 3, overfishing	0	0	0	0	0	0	0	0	0	0	0	1	0	0	0
Species A, Basin 3, dam	0	0	0	0	0	0	0	0	0	0	0	0	1	0	0
Species A, Basin 3, water pollution	0	0	0	0	0	0	0	0	0	0	0	0	0.29	0.57	0.14
Species A, Basin 3, climate change	0.02	0.01	0.17	0.07	0.03	0.01	0.01	0.10	0.05	0.03	0.03	0.01	0.07	0.34	0.05
Species B, Basin 1, dam	0	0	1	0	0	0	0	0	0	0	0	0	0	0	0
Species B, Basin 2, dam	0	0	0	0	0	0	0	1	0	0	0	0	0	0	0
Species B, Basin 2, climate change	0.02	0.01	0.17	0.07	0.03	0.01	0.01	0.10	0.05	0.03	0.03	0.01	0.07	0.34	0.05

Table S11. Numerical example for matrices $\mathbf{C_{ag}}$ and $\mathbf{C_{ag}}$ weighted by species range and human influence, following the example given in **Tabs S8.1-S8.3**. The elements of matrix $\mathbf{C_{ag}}$ sum up to *S* sum up to S basin/species/cause sub-records.

$\mathbf{C_{ag}}$ matrix, accumulation of causes Total: 5 species-basin-cause sub-records	Basin 1					Basin 2					Basin 3				
	Grain	Fishing	Electricity	Industry	Service	Grain	Fishing	Electricity	Industry	Service	Grain	Fishing	Electricity	Industry	Service
Species A, Basin 1, overfishing	0	1	0	0	0	0	0	0	0	0	0	0	0	0	0
Species A, Basin 2, climate change	0.02	0.01	0.17	0.07	0.03	0.01	0.01	0.10	0.05	0.03	0.03	0.01	0.07	0.34	0.05
Species A, Basin 3, ov/da/wp/cc	0.02	0.01	0.17	0.07	0.03	0.01	0.01	0.10	0.05	0.03	0.03	1.01	1.35	0.91	0.19
Species B, Basin 1, dam	0	0	0	0	0	0	0	1	0	0	0	0	0	0	0
Species B, Basin 2, da/cc	0.02	0.01	0.17	0.07	0.03	0.01	0.01	1.10	0.05	0.03	0.03	0.01	0.07	0.34	0.05

**Weighting variables**			
Range of species A	62%	6%	32%
Range of species B	36%	64%	0%
human influence index	19.1	15.7	20.3
**Comprehensive weights**			
Species A	11.84	0.94	6.50
Species B	6.88	10.05	0.00

Weighted $\mathbf{C_{ag}}$ matrix, accumulation of causes Total: 5 species-basin-cause sub-records	Basin 1					Basin 2					Basin 3				
	Grain	Fishing	Electricity	Industry	Service	Grain	Fishing	Electricity	Industry	Service	Grain	Fishing	Electricity	Industry	Service
Species A, Basin 1, overfishing	0	11.842	0	0	0	0	0	0	0	0	0	0	0	0	0
Species A, Basin 2, climate change	0.20	0.06	2.02	0.81	0.40	0.01	0.00	0.10	0.05	0.03	0.22	0.07	0.44	2.22	0.33
Species A, Basin 3, ov/da/wp/cc	0.20	0.06	2.02	0.81	0.40	0.01	0.00	0.10	0.05	0.03	0.22	6.56	8.80	5.93	1.26
Species B, Basin 1, dam	0	0	0	0	0	0	0	10.05	0	0	0	0	0	0	0
Species B, Basin 2, da/cc	0.12	0.04	1.17	0.47	0.23	0.14	0.05	11.08	0.51	0.27	0	0	0	0	0

Table S12. Numerical example for matrices **R** and $\mathbf{R_{ag}}$, following the example given in **Tabs S8.1 and S8.4**. The elements of matrices **R** and $\mathbf{R_{ag}}$ sum up to *R* basin/species threat records.

**R** matrix, normalisation of each record	Basin 1					Basin 2					Basin 3				
Total: 5 species-basin-cause sub-records	Grain	Fishing	Electricity	Industry	Service	Grain	Fishing	Electricity	Industry	Service	Grain	Fishing	Electricity	Industry	Service
Species A, Basin 1, overfishing	0	1	0	0	0	0	0	0	0	0	0	0	0	0	0
Species A, Basin 2, climate change	0.03	0.01	0.29	0.12	0.06	0.00	0.00	0.01	0.01	0.00	0.03	0.01	0.06	0.32	0.05
Species A, Basin 3, ov/da/wp/cc	0.01	0.00	0.08	0.03	0.02	0.00	0.00	0.00	0.00	0.00	0.01	0.25	0.33	0.22	0.05
Species B, Basin 1, dam	0	0	0	0	0	0	0	1	0	0	0	0	0	0	0
Species B, Basin 2, da/cc	0.01	0.00	0.08	0.03	0.02	0.01	0.00	0.79	0.04	0.02	0	0	0	0	0

$\mathbf{R_{ag}}$ matrix, accumulation of records	Basin 1					Basin 2					Basin 3				
Total: 5 species-basin-cause sub-records	Grain	Fishing	Electricity	Industry	Service	Grain	Fishing	Electricity	Industry	Service	Grain	Fishing	Electricity	Industry	Service
Species A	0.04	1.01	0.37	0.15	0.07	0.00	0.00	0.02	0.01	0.00	0.04	0.26	0.40	0.54	0.10
Species B	0.01	0.00	0.08	0.03	0.02	0.01	0.00	1.79	0.04	0.02	0.00	0.00	0.00	0.00	0.00
